# How Sensitive Is the Human Visual System to the Local Statistics of Natural Images?

**DOI:** 10.1371/journal.pcbi.1002873

**Published:** 2013-01-24

**Authors:** Holly E. Gerhard, Felix A. Wichmann, Matthias Bethge

**Affiliations:** 1 Computational Vision and Neuroscience Group, Max Planck Institute for Biological Cybernetics, Tübingen, Germany; 2 Werner Reichardt Centre for Integrative Neuroscience, University of Tübingen, Tübingen, Germany; 3 Bernstein Center for Computational Neuroscience, Tübingen, Germany; 4 AG Neuronale Informationsverarbeitung, Mathematisch-Naturwissenschaftliche Fakultät, Eberhard Karls Universität Tübingen, Tübingen, Germany; 5 Abteilung Empirische Inferenz, Max-Planck-Institut für Intelligente Systeme, Tübingen, Germany; 6 Institute of Theoretical Physics, Eberhard Karls Universität Tübingen, Tübingen, Germany; Northwestern University, United States of America

## Abstract

A key hypothesis in sensory system neuroscience is that sensory representations are adapted to the statistical regularities in sensory signals and thereby incorporate knowledge about the outside world. Supporting this hypothesis, several probabilistic models of local natural image regularities have been proposed that reproduce neural response properties. Although many such physiological links have been made, these models have not been linked directly to visual sensitivity. Previous psychophysical studies of sensitivity to natural image regularities focus on global perception of large images, but much less is known about sensitivity to local natural image regularities. We present a new paradigm for controlled psychophysical studies of local natural image regularities and compare how well such models capture perceptually relevant image content. To produce stimuli with precise statistics, we start with a set of patches cut from natural images and alter their content to generate a matched set whose joint statistics are equally likely under a probabilistic natural image model. The task is forced choice to discriminate natural patches from model patches. The results show that human observers can learn to discriminate the higher-order regularities in natural images from those of model samples after very few exposures and that no current model is perfect for patches as small as 5 by 5 pixels or larger. Discrimination performance was accurately predicted by model likelihood, an information theoretic measure of model efficacy, indicating that the visual system possesses a surprisingly detailed knowledge of natural image higher-order correlations, much more so than current image models. We also perform three cue identification experiments to interpret how model features correspond to perceptually relevant image features.

## Author Summary

Several aspects of primate visual physiology have been identified as adaptations to local regularities of natural images. However, much less work has measured visual sensitivity to local natural image regularities. Most previous work focuses on global perception of large images and shows that observers are more sensitive to visual information when image properties resemble those of natural images. In this work we measure human sensitivity to local natural image regularities using stimuli generated by patch-based probabilistic natural image models that have been related to primate visual physiology. We find that human observers can learn to discriminate the statistical regularities of natural image patches from those represented by current natural image models after very few exposures and that discriminability depends on the degree of regularities captured by the model. The quick learning we observed suggests that the human visual system is biased for processing natural images, even at very fine spatial scales, and that it has a surprisingly large knowledge of the regularities in natural images, at least in comparison to the state-of-the-art statistical models of natural images.

## Introduction

**Figure 1:**
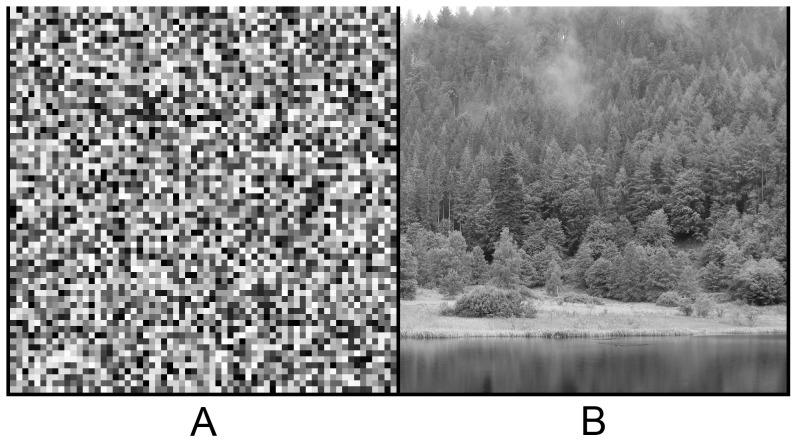
Different kinds of images. **A.** A white noise image free of spatial correlations between pixel gray values. **B.** A natural image. In the present work, we study sensitivity to local regularities in natural images.

We operate in a world exhibiting statistical regularities. In a very different universe where every point in space were independent from all others, white noise images (Figure 1A) would be common place. Of course, our world appears much more structured (Figure 1B). It contains objects with smoothly and slowly varying surface features, which make nearby parts of space appear similar. If there is such structure in the world, visual representations in the brain should take these correlations into account, as stated by the efficient coding hypothesis [1], [2]. One way to test this idea is to build models that specify a probability density function over the space of natural images and compare the resulting model features with known physiological properties of the visual system. Similarities between model features and neural properties are frequently taken as evidence that the visual system has similarly acquired knowledge of the natural image distribution: bandpass filtering [3], [4], orientation selectivity [5], [6], divisive normalization [7]–[10], and complex cell pooling [11]. These findings are at least consistent with the idea that the visual system is adapted to the statistical regularities in natural images. In the present work, we take a different approach, which is to measure the visual sensitivity of human observers to statistical regularities in natural images.

Much of the previous psychophysical work using natural images focuses on full size images and sensitivity to measures derived from the Fourier transform. For example, natural images show a 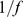 fall-off in their amplitude spectra [12]. When amplitude spectra are similar to those of natural images, human observers perform better on a variety of discrimination tasks [13]–[17]. Other studies have explored sensitivity to properties encoded in the Fourier phase spectrum with varied approaches and results [18]–[25]. The phase spectrum globally encodes shape information [26]. Fewer psychophysical studies have focused on sensitivity to local natural image regularities. Observers can predict extremely local image values better in natural than in random images [27], indicating that the visual system also makes use of local natural image regularities. The texture modeling literature has also established several local image statistics as perceptually important for successful reproduction of natural textures [28]–[30].

In this work, we measure human sensitivity to local regularities in natural images using probabilistic models learned on patches of natural images, which allows us to construct stimuli that pit the full range of local natural image regularities against a limited range controlled by a model. In so doing, we test the efficacy of different kinds of natural image models in capturing perceptually prominent image features. Depending on the nature of a model's assumptions, it captures a particular degree of the statistical regularities present in natural images, which can be estimated via model likelihood. The five probabilistic models we utilize for stimulus generation have been evaluated quantitatively using cross-validated model likelihood estimates (Table 1) and represent a range of advances in capturing natural image regularities, e.g. [3]–[11], [31]–[33]. They can also be grouped into classes that differ in characteristic features related to primate visual physiology (Table 1).

**Table 1:** Natural image model features and likelihood estimates.

	BF	OS	DN	CP	Likelihood	References
					(bits/pixel)	
**RND/PCA/Whitening**	x				2.7	[8], [43]
**ICA**	x	x			2.9	[8], [43]
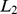 **-spherical**	x		x		3.05	[8]–[10]
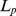 **-spherical**	x	x	x		3.17	[62]
**MEC with** 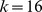	x	x	x	x	3.3	[33]

The natural image models we tested along with the neural response properties they mimic: “BF” is bandpass filtering, “OS” is orientation selectivity, “DN” is divisive normalization, and “CP” is complex cell pooling. We also show cited likelihood estimates. MEC is the mixture of elliptically contoured distributions model [33]. All models are described in detail in the “Models Tested” section. Higher likelihood indicates that a model captures more of the regularities present in natural images than a model with lower likelihood.

**Figure 2:**
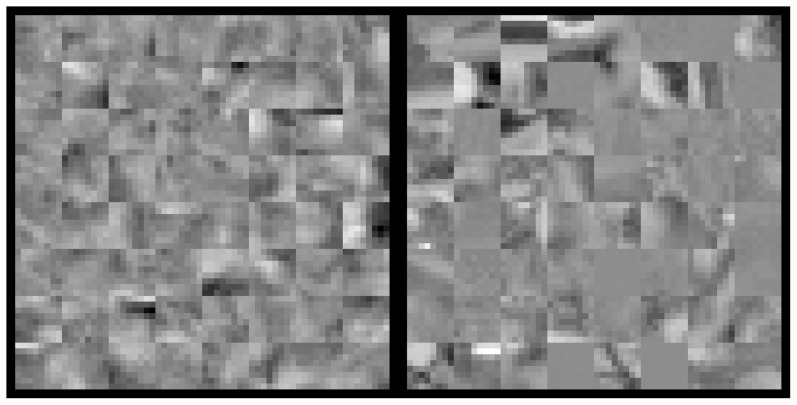
Example stimulus. The left texture contains model samples, and the right texture contains only true natural image samples. Each texture is a square tiling of 64 samples, where each sample is 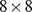 pixels in size. The observer's task is to indicate the texture made only of natural image samples. Feedback was given, and a short training sequence was performed before every experiment.

In our paradigm, observers perform a discrimination task where model generated samples are pitted against true natural image patches. We tile the two sets of image patches into separate textures, such as are shown in Figure 2, and ask the observer to select the texture of true natural image patches. The model samples are generated by redistributing the natural image content under the specific model assumptions, which preserves the patches' joint probability under the model but destroys higher-order regularities that the model assumptions fail to capture. Following Julesz's original conjecture [34], above chance performance results only when the observer can make use of those additional higher-order regularities present in the natural image patches.

The benefits of our approach are two-fold. First, by comparing the natural image models in a psychophysical framework, we complement the model comparisons based on likelihoods by a rigorous evaluation of how well the different models are able to capture perceptually relevant features. Second, we learn about the biases of the human visual system by examining whether differences in difficulty between the models relate to their statistical properties. Because our experiments are relatively short in duration (each less than 90 minutes), and natural images contain very complex regularities, fast learning results only if the human visual system is biased to process natural images [35].

In the first experiment, we measure discrimination performance for all models using grayscale patches that contain a number of potential cues. We find that human observers achieve above chance performance whenever image patches are at least 5 by 5 pixels in size and that performance depends on model likelihood, suggesting the human visual system is optimized for processing natural image regularities even at a small scale. We cannot tell directly from a single experiment how the human visual system is biased for this task. Previous psychophysical studies using images with controlled regularities have identified several image statistics to which the human visual system is sensitive, including luminance histogram features [36], and structural shape-related features [37]–[40]. Furthermore, pixel histograms and other Fourier-based features are known to be important in the representation of natural textures [30]. In three cue identification experiments, we examine the extent to which these kinds of features explain the discriminability of our models from the natural image distribution.

## Results

### Measuring sensitivity to local natural image regularities

To create stimuli with controlled regularities, we start with a set of natural image patches and generate a set of model patches equal in joint probability under the model. The patches are therefore matched in terms of the regularities captured by the model. The generation process makes use of the model assumptions critical for avoiding the curse of dimensionality: we shuffle the content of the natural image patches by applying the symmetry or independence assumptions of the model. The two sets of image patches then comprise a single discrimination trial.

**Figure 3:**
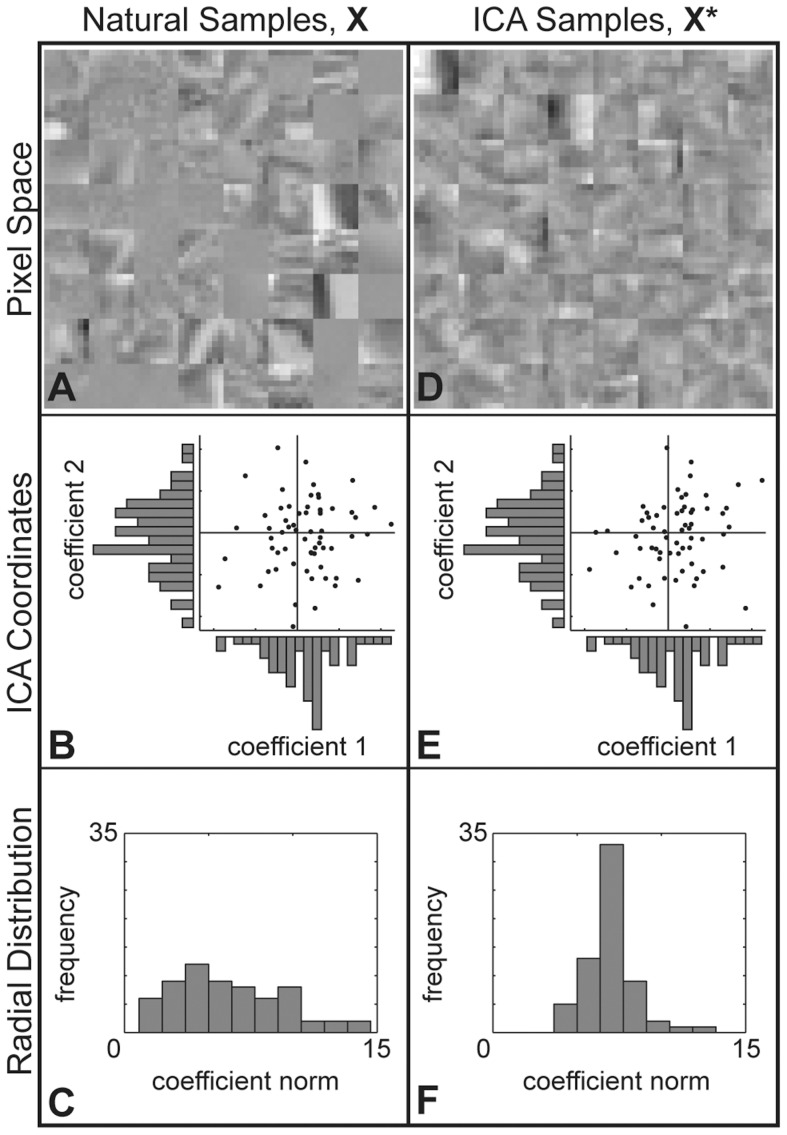
Generating model samples using ICA. **A.** A set of 64 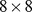 pixel natural image patches, 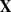. **B.** The coefficients of the first two (non-DC) ICA components are plotted against each other for all 64 patches along with their marginal distributions. **C.** Histogram of the 64 patches' norms in the ICA basis. **D.** To apply the ICA independence assumption to 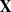, we shuffle the ICA coefficients across samples separately for each component. Shown are the resulting matched model patches, 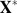. **E.** The coefficients of the first two (non-DC) ICA components of 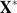. The marginal distributions are the same as those of 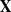 shown in **B**. **F.** Histogram of the coefficient norms of the 64 patches in 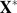. Applying the ICA assumption has changed the radial distribution so that the variance is much lower than that of the original distribution shown in **C**.

To illustrate the image generation process, we now step through an example of applying the independence assumption to a set of natural image patches, 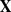, cut from random locations in various photographs of a natural image database [41]. Figure 3A shows a set of 64 such patches. Consider the independent components analysis model (ICA) [6]. Learning the model on a very large database of natural image samples, 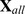, yields an ICA basis. To apply the independence assumption to 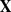 and generate a new set 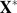 matched in joint-probability under ICA, we first transform 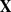 into ICA coordinates and then shuffle the values of each coordinate separately across the patches, which preserves the marginal distributions of the coordinates. The resulting ICA-matched patches 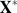 are shown in Figure 3D. We plot the first two non-DC components of 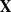 in Figure 3B and of 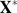 in Figure 3E with their marginal distributions. As shown, the marginal distributions are preserved after applying the ICA independence assumption. The radial distribution, however, has changed as shown in Figure 3F versus Figure 3C, indicating that the independence assumption of ICA is not fulfilled for natural images.

**Figure 4:**
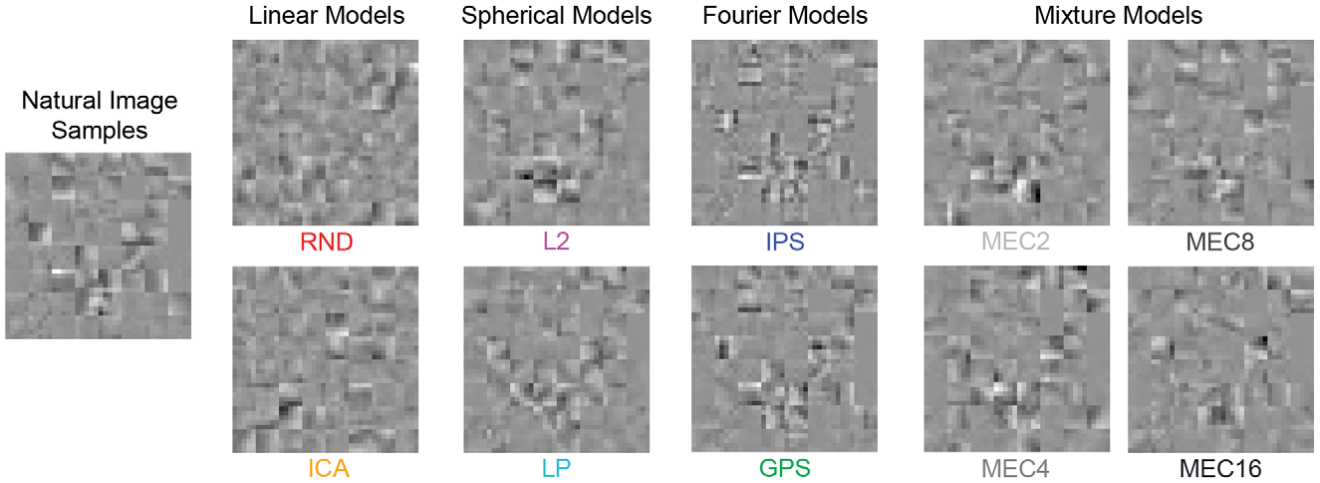
Image patch examples from Experiment 1. In Experiment 1, we tested six models in one session (RND, ICA, L2, LP, IPS, GPS) and the four mixture models in a separate session (MEC2, MEC4, MEC8, MEC16). Shown are example textures for each model. The 64 samples comprising each model texture are matched to the 64 natural image samples shown on the left. Patch size here is 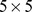 pixels. On any single trial, observers viewed only one set of natural image samples and one set of model samples (e.g. as shown in Figure 2).

The image patches in 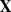 and 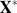 are then used as stimuli for a discrimination task. In each trial, 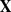 and 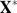 are presented simultaneously on a black background, each shown as a texture made by tightly tiling the 64 image patches (e.g. Figure 4). The observer's task is to indicate which texture is composed of true natural image patches.

To measure the discriminability of a particular model from the natural image distribution, we perform several trials with different 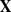. If the human visual system were sensitive only to the regularities described by the model, discriminability should be at chance. Above chance performance indicates sensitivity to the natural image regularities not captured by the model. To increase the sample size of natural images contributing to each discriminability estimate, we will pool estimates over observers and trials since each trial uses a unique set of natural image patches, 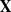, sampled uniformly across a very large database of natural images.

In the following section, we provide detailed descriptions of the models tested, their shuffling procedures, and proofs that joint probability is matched after shuffling.

### Models tested

All models were learned on log-luminance natural images from the Van Hateren natural image database [41]. We used log-luminance values because uniform changes in logarithmic luminance are equally detectable following the Weber-Fechner law. The log transform is also a standard procedure in natural image modeling because it decreases the asymmetry of the natural image luminance distribution, making it easier to model the higher order regularities.

From the model capturing the fewest regularities to the model capturing the most, we test: 1) a random second-order model capturing only second-order correlations (RND), 2) the independent components analysis model (ICA), 3) the 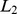-spherical model (L2), 4) the 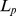-spherical model (LP), and 5) the mixture of elliptically contoured distributions model with four levels of mixtures (MEC with 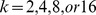) [33]. Roughly speaking, MEC is able to capture similar correlations like the Karklin & Lewicki model [11], or the mixture of Gaussian scale mixtures model [32], yet MEC uses hard clustering to partition the natural image distribution which we make use of in the stimulus generation process. Thus, each cluster is described exclusively by a zero-mean elliptically contoured distribution with its own covariance.

We first discuss RND and ICA, the two linear models of natural images that we test. A linear model is defined to have statistically independent components after a linear transformation of the pixel values. The RND model consists of a set of un-oriented “pink noise” filters that capture only the covariance of natural image gray values, and ICA consists of a set of oriented filters additionally optimized for higher-order correlations. In the following, we will use vectorized image patch notation to describe a set of natural image patches 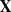, where 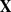 is a 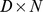 matrix of 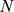 patches containing 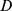 pixels each. We use lower case 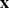 to denote a single image patch in 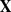. A linear model is fully specified by its filter matrix 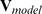. To obtain the coefficients of a single patch in the representation space of that model, we compute 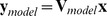. The joint probability of a set of 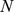 image patches, 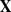 is given by
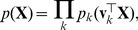
(1)where 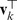 denotes the 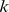-th row vector of the filter matrix 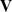 and is one of many filters of the linear transformation. In general, it holds that 
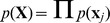
 since the patches are drawn independently from the same distribution. Therefore, we obtain
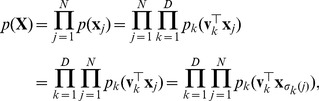
(2)where 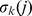 denotes an arbitrary permutation over the patches in 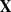. As this equation shows–-by the commutativity of products—we can generate a new, equally probable set of patches 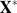 by shuffling the 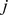-indicies.

The other three natural image models we test, L2, LP, and MEC, do not assume independence after the linear transformation. Instead they assume that after some linear transformation, the natural image distribution obeys certain symmetry assumptions and can be transformed into a factorial representation of independent components only by non-linear transformations.

The 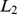-spherically symmetric model is a generalization of Gaussian scale mixtures [31] which assumes spherical symmetry after whitening and can be made factorial by radial Gaussianization [9], [10]. Due to the spherical symmetry, the model is only sensitive to the power spectrum of the filters but insensitive to their detailed shape–-i.e., the model is sensitive to changes in 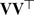 and thus ignores changes in 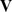 that result from an orthogonal mapping. Thus, like for RND, we chose random filter shapes for L2 akin to pink noise that capture the second-order correlations but have no specific shape otherwise.

The 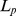-spherical model is a generalization of the 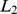-spherical model which allows one to optimize the detailed filter shapes for additional higher-order correlations. While the density, 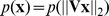, of the 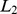-model is a function of the 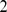-norm and thus invariant under arbitrary rotations of 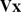, the density of the 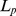-model, 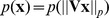, is a function of the 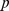-norm, 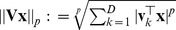, which is invariant only under permutations of the coordinates. Optimizing the 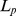-model for the van Hateren dataset [41], which we used to test the models, yields 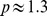 and the same oriented filter shapes as in the ICA model [10]. Also, the 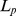-spherical distribution can be made factorial by using radial factorization instead of radial Gaussianization [10].

The joint probability of a set of natural image patches 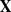 under either the 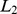
- or 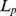-model can be written as
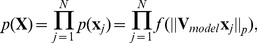
(3)where in the case of L2, the filter matrix 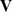 is the same as that for RND, and in the case of LP, 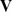 is the same as that for ICA.

We now show why permutation of the model's representation coordinates, 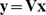, within a patch preserves the patch's norm. If 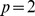, then 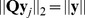 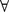 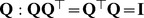 because 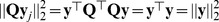. If 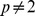, then 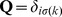 where 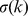 is a random permutation of the coordinate indices and 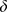 is the Kronecker delta, which equals 1 when 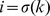 and zero otherwise. After permuting the coefficients within an image patch, we denote the new image patch as 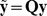. The 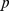-norm of 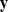 is 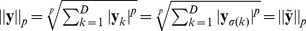. Therefore, permuting the coordinates of a patch obtained from 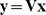 preserves the norm in the model's representation space, and the set of generated image patches 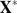 is equally probable to 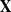 following Equation 3.

Because MEC uses hard clustering to obtain non-overlapping clusters, we model each cluster by its own 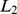-model each using a different whitening transform. Thus, we can simply apply the 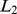-norm symmetry to each patch once it has been transformed into the appropriate representation of its cluster.

The models vary in complexity which is also reflected by the transforms necessary to obtain a factorial representation. The properties of these redundancy reduction transforms can be related to primate visual physiology (Table 1). Linear models are linked to linear response properties that can be further divided into power and phase spectral information. RND captures the *power* spectral properties that are common to center-surround models of retina and LGN [3], [4] and the PCA and ICA models [42], but it does not reproduce the more special filter shape properties determined by the differences between the models in the *phase* spectra. RND is therefore useful as a baseline model to disentangle the contribution of matching the second-order statistics from more specific receptive field properties. At the other extreme, ICA optimizes the more specific filter shape properties determined by the phase spectra with respect to higher-order correlations [6], making ICA the best possible linear model. (The center-surround model or PCA model constitute intermediate cases because their filter shape properties are better matched to natural image statistics than RND but less matched than ICA [43].) The difference in performance between RND and ICA thus reflects the maximal effect among linear models that the phase spectral properties of filter shapes can have.

L2 and LP are nonlinear models from the class of 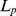-spherical models, which are related to contrast gain control [7]–[10]. Because L2 and LP use the same filters as RND and ICA respectively, again the difference in performance between L2 and LP represents the maximal effect that the filter shapes can have beyond matching the power spectra.

The mixture model also captures oriented features and represents different classes of images separately, and it is related to the model of Karklin and Lewicki, another mixture model learned on the natural image distribution which showed complex cell-like pooling properties [11].

Another important reason why we selected this set of models to test is because cross-validated likelihood estimates have already been reported in the literature for each of them, which we list with citations in Table 1. All of these likelihoods were estimated in the most conservative way, where test sets and training sets of the same size were used, and the difference in likelihood between the training and test sets was tiny.

We also test two Fourier “models” of natural image patches. Although we do not have their likelihood estimates, these models are intended as comparisons where patch-based Fourier statistics are isolated. Both preserve the amplitude spectra of the patchwise Fourier transforms of each patch in 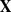, which carries most of the image appearance information for small patches [44]. We test independent phase scrambling (IPS), in which we preserve the patchwise power spectra and randomize the patchwise phase spectra, and we test global phase scrambling (GPS), which preserves all correlations between phases and between amplitudes yet destroys dependencies between the two.

### Scale of local regularities

Natural image patches were sampled from a database of grayscale photographs of outdoor scenes where 1 pixel equals approximately 2 minutes of arc [41]. Discriminability was measured for different model generated stimuli at the following patch sizes: 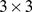, 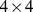, 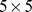, and 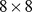 pixels, corresponding to a range of 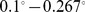 in the original photographs. We therefore examined regularities occurring at a very fine scale in natural images, one above yet nearing the human resolution limit. We always magnified the patches on the screen because we were interested in whether observers can discriminate the regularities present in natural images at this fine scale and not in whether acuity was good enough for the task at this scale.

### Experiment 1: Grayscale stimuli with many potential cues

In Experiment 1, observers discriminated grayscale natural image samples from model samples, and the stimuli included all potential cues. A pair of example stimulus textures is shown in Figure 4. In one session, 16 observers performed the task for RND, ICA, L2, LP, IPS, and GPS at patch sizes 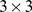, 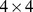, and 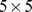 pixels. In a second session, 12 observers performed the task for the four versions of MEC with 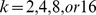. Because MEC is among the best in terms of likelihood (Table 1), we additionally included 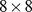 pixel patches. Each observer completed 30 trials for each model at each patch size for a total of 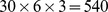 trials in session one and 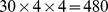 trials in session two.

**Figure 5:**
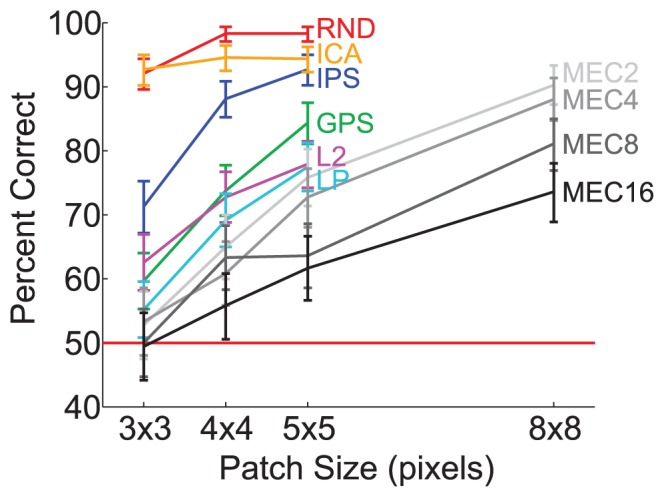
Experiment 1 results. Discriminability estimates with 95% binomial confidence intervals are shown by model as a function of patch size, where data are pooled over subjects. Sixteen subjects participated in session one with RND, ICA, L2, LP, IPS, and GPS, and 12 participated in session two with the MEC models. Each subject performed 30 test trials per data point in the plot. Therefore, each data point for session one is based on 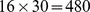 trials, and each for session two is based on 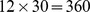 trials.

Average discrimination performance is plotted in Figure 5 as a function of patch size with 95% binomial confidence intervals. Discriminability estimates are also printed in Table 2 with stars to indicate 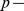
values. MEC 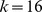 was the most difficult model to discriminate, and only the MEC models brought performance to chance (with 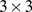 pixel patches). Observers were near ceiling with the linear models, RND and ICA, achieving respectively 96% and 94% correct on average. The spherical models, L2 and LP, were more difficult, with average discriminability dropping to 71% and 67% correct respectively. IPS and GPS were roughly between the linear and spherical models in terms of difficulty, with average discriminability at 84% and 73% correct respectively. Overall, the large proportion of data points above chance indicates that the human visual system is highly sensitive to the local features of natural images, even to the higher-order regularities the best models fail to capture. Discriminability estimates were always significantly above chance for 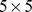 pixel patches and larger, indicating that no model sufficiently captured all the prominent features for patches larger than 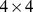 pixels in size.

**Table 2:** Experiment 1 average discriminability for all models.

	Patch size (pixels)			
	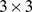	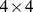	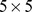	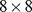
**RND**	92.1 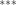	98.3 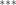	98.3 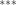	-
**ICA**	92.7 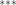	94.6 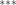	94.4 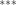	-
**IPS**	71.3 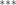	88.1 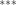	92.7 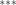	-
**GPS**	59.7 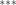	73.8 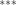	84.4 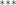	-
**L2**	62.6 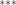	72.8 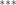	77.9 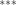	-
**LP**	55.2 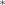	69.2 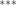	77.5 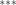	-
**MEC,** 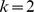	52.8	65.0 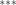	75.8 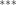	90.3 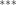
**MEC,** 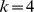	53.3	60.8 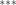	72.8 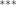	88.1 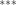
**MEC,** 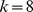	50.0	63.3 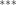	63.6 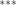	81.1 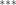
**MEC,** 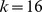	49.4	55.8^*^	61.7 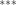	73.6 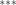

Average percent corrects are listed for each model at each patch size tested. 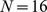 subjects for the first six models, and 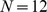 subjects for the MEC models. In starred conditions the null hypothesis that performance was at chance (50%) can be rejected at the 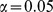 level (*), the 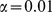 level (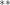), or the 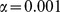 level (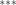). 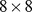 pixel patches were tested only for the MEC models.

**Figure 6:**
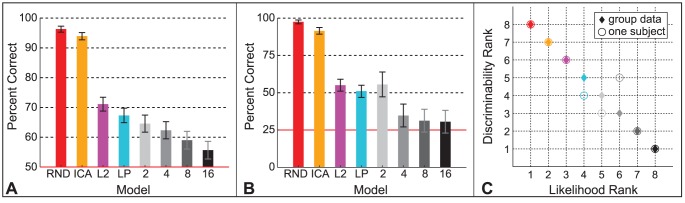
Model discriminability and likelihood. **A.** Discriminability estimates with 95% binomial confidence intervals plotted in order of increasing model likelihood. Data is pooled over subjects and patch sizes 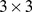, 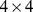, and 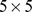 pixels. Each data point for RND, ICA, L2, and LP contains 1,440 trials, and 1,080 trials for the MEC models. MEC models are identified by the number of mixtures. Chance performance was 50%. **B.** Discriminability estimates with 95% binomial confidence intervals for one subject who performed 5 sessions of a four alternative choice version of the experiment. Each data point for RND, ICA, L2, and LP contains 576 trials, and 144 for the MEC models. Chance was 25%. **C.** Discriminability ranks of the models from most difficult to easiest are plotted against likelihood ranks from lowest likelihood to highest. Diamonds show group average data from **A**, and circles show the individual subject's data from **B**. The group data contain more trials and show a clear decrease in discriminability with increased likelihood. The same order is shown in the individual subject data within the range of the 95% confidence intervals, which overlap for L2, LP, and MEC 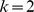.

To examine how performance was related to model likelihood, we plotted model discriminability in order of increasing model likelihood, based on the likelihood estimates in Table 1 where RND is equivalent to PCA, and MEC's likelihood increases with more mixtures. (We do not include the Fourier models here as they are not probabilistic models and hence do not have likelihoods.) Each bar in Figure 6A is one model's discriminability with 95% binomial confidence intervals for data pooled over subjects and patch sizes 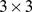, 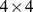, and 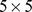 pixels. RND, ICA, L2, and LP estimates are based on 1,440 trials each, and MEC models on 1,080 trials each. Discriminability decreases as model likelihood increases.

We analogously examined the data of a single subject, plotted in Figure 6B. The subject performed 4,032 trials of a more sensitive version of the experiment (chance = 25%) in 4 sessions with RND, ICA, L2, LP, IPS, and GPS and one session with the MEC models. In Figure 6B, estimates for RND, ICA, L2, and LP are based on 576 trials each, and MEC estimates on 144 trials each. Within the range of the 95% binomial confidence intervals, which overlap for L2, LP, and MEC 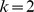, theses data show the same pattern of decreased discriminability with increased model likelihood.

In Figure 6C, we plot the model ranks in terms of discriminability against the model ranks in terms of likelihood for all the data points in Figure 6A and B. The pattern shows that discriminability decreases as model likelihood increases.

Feedback was provided throughout the experiment, so we analyzed the data for learning by splitting the data in half over time and comparing discriminability estimates across the two halves. If the 95% binomial confidence intervals of the two estimates do not overlap, there may have been learning (or anti-learning). Binomial confidence intervals assume trial independence and therefore underestimate confidence interval width in the case that subjects' behavior was non-stationary [45], so the test we report is biased away from false negatives and thus rather over sensitive to learning. We applied this test for each model separately with data pooled over subjects and patch sizes. In the 2AFC version of the experiment with 16 subjects, the only significant effect was for the ICA model: discriminability increased by 4% from 91% to 95% correct. In the 4AFC version of the experiment with 1 subject, discriminability with ICA also improved from 87% to 96% correct and with IPS from 70% to 82% correct.

### Experiment 2: Luminance histogram cues only

**Figure 7:**
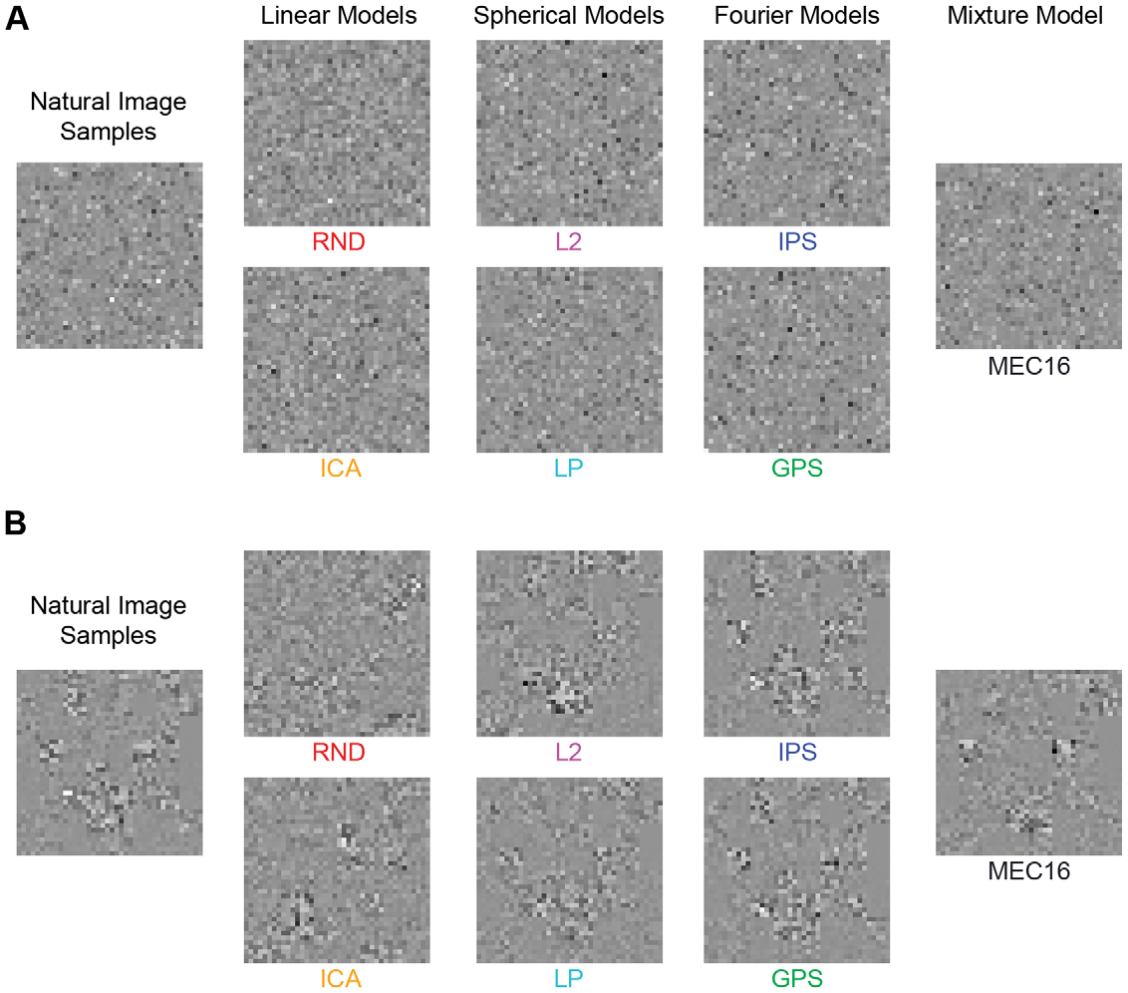
Experiment 2 texture scrambles. Here we show example textures for each model tested in Experiment 2: RND, ICA, L2, LP, IPS, GPS, and MEC16. Both **A** and **B** are scrambled versions of the corresponding model stimuli shown in Figure 4. On any single trial the observer viewed only one texture based on natural image samples and one texture based on samples from a single model. **A.** Global scrambles, where the pixels of each texture were scrambled as a final post-processing step. **B.** Sample scrambles, where the pixels of each image patch were scrambled individually to preserve variations in luminance histograms across samples.

Human observers can discriminate textures on the basis of three mechanisms sensitive to luminance histogram features [36]. We therefore hypothesized that luminance histogram differences between natural samples and model samples were a prominent cue. We tested this hypothesis in Experiment 2, where we compared two new manipulations to performance in Experiment 1, whose stimuli contained several potential cues, including both shape and luminance features. We will refer to them as the “unperturbed” stimuli. The two new manipulations used pixel-scrambling, which was applied to the unperturbed stimuli as a final post-processing step before presenting the textures. In one condition we permuted the pixels globally within each texture to produce “global scrambles” (Figure 7A). In the second condition, we permuted pixels within each unperturbed image patch separately to produce “sample scrambles” (Figure 7B).

We tested all models from Experiment 1 except that we tested only the best MEC model, MEC with 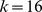, for which we excluded 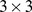 pixel patches since observers were at chance in Experiment 1 at that size. All other aspects of Experiment 2 were identical to Experiment 1. Three observers participated. Each completed 30 trials per patch size per model per condition for a total of 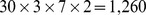 trials.

**Figure 8:**
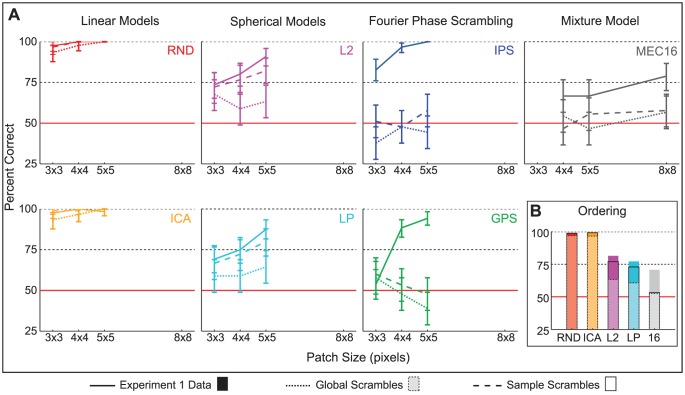
Experiment 2 results. **A.** Discriminability estimates with 95% binomial confidence intervals are shown by model as a function of patch size. Three subjects participated, and each performed 30 test trials per model per patch size per condition, so each data point is based on 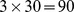 trials. We did not measure discriminability for MEC 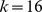 with 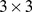 pixel patches as observers were at chance with them in Experiment 1. The solid line shows these observers' data in Experiment 1, i.e. with unperturbed stimuli, the dotted line shows performance for global scrambles, and the dashed line for sample scrambles. **B.** Discriminability estimates averaged over patch size for each model are plotted in order of increasing likelihood. The colored bars are the data from Experiment 1, the translucent bars with dashed edges are for the global scrambles, and the bars with solid edges are for the sample scrambles. In all three conditions, the ordering is the same: higher likelihood is linked with lower discriminability.

The results are shown in Figure 8A as percent corrects pooled over the 3 observers with 95% binomial confidence intervals. Solid lines are these observers' discriminability estimates for the unperturbed stimuli (from Experiment 1). Dotted lines are for the global scrambles, and long dashed lines are for the sample scrambles. Discriminability of the linear models, RND and ICA, was unaffected by both types of pixel scrambling and remained near ceiling. This indicates that luminance histogram cues were sufficient for observers to discriminate the unperturbed RND and ICA samples from natural samples. Furthermore, ceiling performance indicates the luminance histogram cues were highly prominent for RND and ICA. With L2 and LP, discriminability dropped close to chance with global scrambles, but there was very little difference between discriminability of sample scrambles and of unperturbed stimuli, which indicates that the L2 and LP models failed to reproduce luminance histogram variations across natural samples. Observers were at chance with both types of scrambles for IPS, GPS, and for MEC 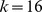.

We also plot discriminability estimates averaged over all patch sizes in order of model likelihood in Figure 8B for each condition separately: colored bars are for the Experiment 1 data, translucent bars with dashed edges for the global scrambles, and transparent bars with solid edges for the sample scrambles. The same ordering in terms of discriminability was found in all conditions and followed the likelihood ordering as in Experiment 1.

The overall results indicate that contrast fluctuations are a highly prominent feature of natural images that is completely lost by linear models and poorly captured by the spherically symmetric models. Of the probabilistic models we tested, only MEC 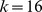 captured the contrast fluctuations so well as to fool the human observer.

Using the learning test reported in Experiment 1, we also analyzed the data for each model separately for the two conditions with data pooled over subjects and patch sizes. There were no significant effects of learning, but discriminability significantly decreased for LP in the global scrambling condition by 10% from 66% to 56% correct.

### Experiment 3: Grayscale shape cues highlighted

**Figure 9:**
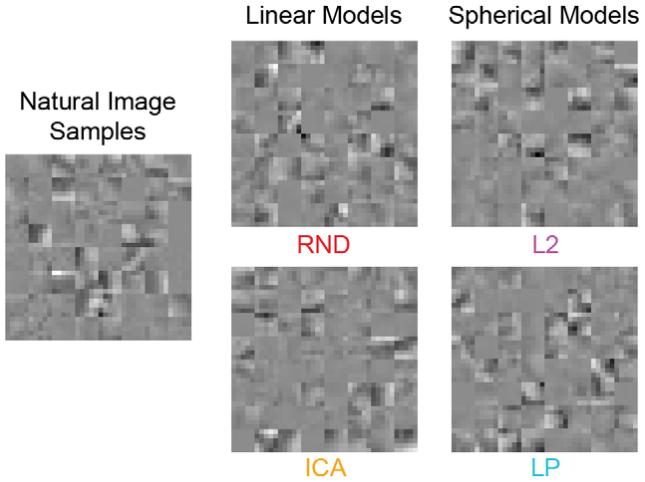
Experiment 3 contrast fluctuation matched model samples. The contrast fluctuations of each model sample set have been artificially matched to the contrast fluctuations across the natural samples by matching the distribution of grayscale pixel norms to that of the natural samples. Each texture is the fluctuation matched version of the corresponding stimulus in Figure 4.

In Experiment 3, we measured sensitivity to the shape content of natural images separately from the effects of the highly prominent contrast fluctuations found in Experiment 2. To this end, we developed a procedure for removing the contrast fluctuation cue by matching the contrast fluctuations across model samples to those in the natural samples on a trial-by-trial basis. An example stimulus is shown in Figure 9. Figure 4 contains the unperturbed version of the same samples.

This manipulation makes the task more difficult, so we modified the task to allow observers to inspect the images as long as they needed while also encouraging them to reply as quickly as possible without sacrificing accuracy. We compare discriminability estimates for the unperturbed version and the contrast fluctuation matched version both run under the same experiment parameters. The experiment was therefore two conditions, which we randomly interleaved in one session. We tested only RND, ICA, L2, and LP. In Experiment 2 we found that MEC, IPS, and GPS perfectly model the contrast fluctuations for observers, indicating that the results of Experiment 1 had already revealed how well these models capture shape information when luminance histograms are well matched. To avoid redundancy and make good use of our observers' time, we therefore excluded them here. We measured performance at patch sizes 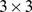, 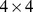, 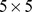, and 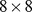 pixels. Nine observers participated. Each observer completed 36 test trials per model per condition per patch size for a total of 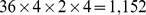 trials. All other experiment details were the same as in Experiment 1. We use ^*^ after model names for the condition where contrast fluctuations were artificially matched to the natural samples since the manipulation alters the probabilistic models.

**Figure 10:**
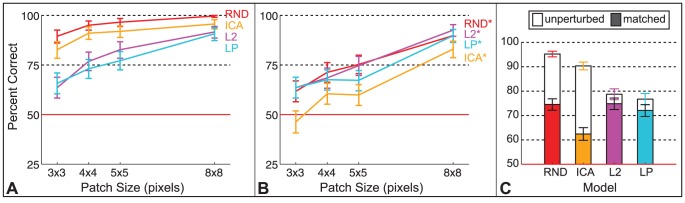
Experiment 3 results. Discriminability estimates are plotted with 95% binomial confidence intervals. Nine subjects participated, and each performed 36 test trials per model per condition per patch size, so each data point in **A** and **B** is based on 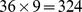 trials. MEC, IPS, and GPS were not included in the experiment because they perfectly captured the contrast fluctuation cue in Experiment 2. **A.** Results from the unperturbed stimulus condition. **B.** Results from the contrast fluctuation matched stimulus condition. **C.** Discriminability estimates pooled over patch sizes and plotted in order of increasing model likelihood. The unfilled bars are for the unperturbed stimulus data in **A**, the filled bars for the data in **B**. As expected the model ordering for the data in **A** are the same as in Experiment 1, but the model ordering changed for the contrast fluctuation matched data, showing that 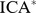 brought performance closest to chance out of all models whereas ICA was near ceiling with the unperturbed stimuli.

Results are shown in Figure 10A for the unperturbed stimuli and in Figure 10B for the contrast fluctuation matched stimuli. We plot the average discriminability over all patch sizes for each model in order of increasing likelihood in Figure 10C, where unfilled bars are for unperturbed stimuli and filled are for matched stimuli. The unperturbed results are similar to the Experiment 1 results. In particular, the discriminability rankings of the models were the same. Surprisingly, the rankings followed a different pattern with contrast fluctuation matched stimuli: estimates were very similar for 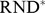, 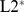, and 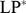, on average 74% correct, and 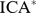 was more difficult than the other models. In fact, the average discriminability of 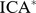, 62% correct averaged over patch sizes, was on par with that of MEC 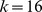 in Experiment 1, 60% correct. 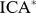 also brought performance to chance for 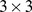 pixel patches, a great improvement over the unperturbed version.

Using the learning test reported in Experiment 1, we also analyzed the data for each model separately for the two conditions with data pooled over subjects and patch sizes. There were no significant effects of learning.

### Experiment 4: Binary images

In Experiment 3, where we highlighted the shape content of natural images, we found a surprising result that the discriminability ranking of the models changed dramatically when the contrast fluctuation cue was removed. To examine the robustness of this effect, we performed a second manipulation focusing on shape content. This time we preserved the statistical properties of the models by using binary images as stimuli, where we thresholded gray values as a final post-processing step before presenting the stimuli. This procedure preserves luminance contours and hence some basic shape content.

**Figure 11:**
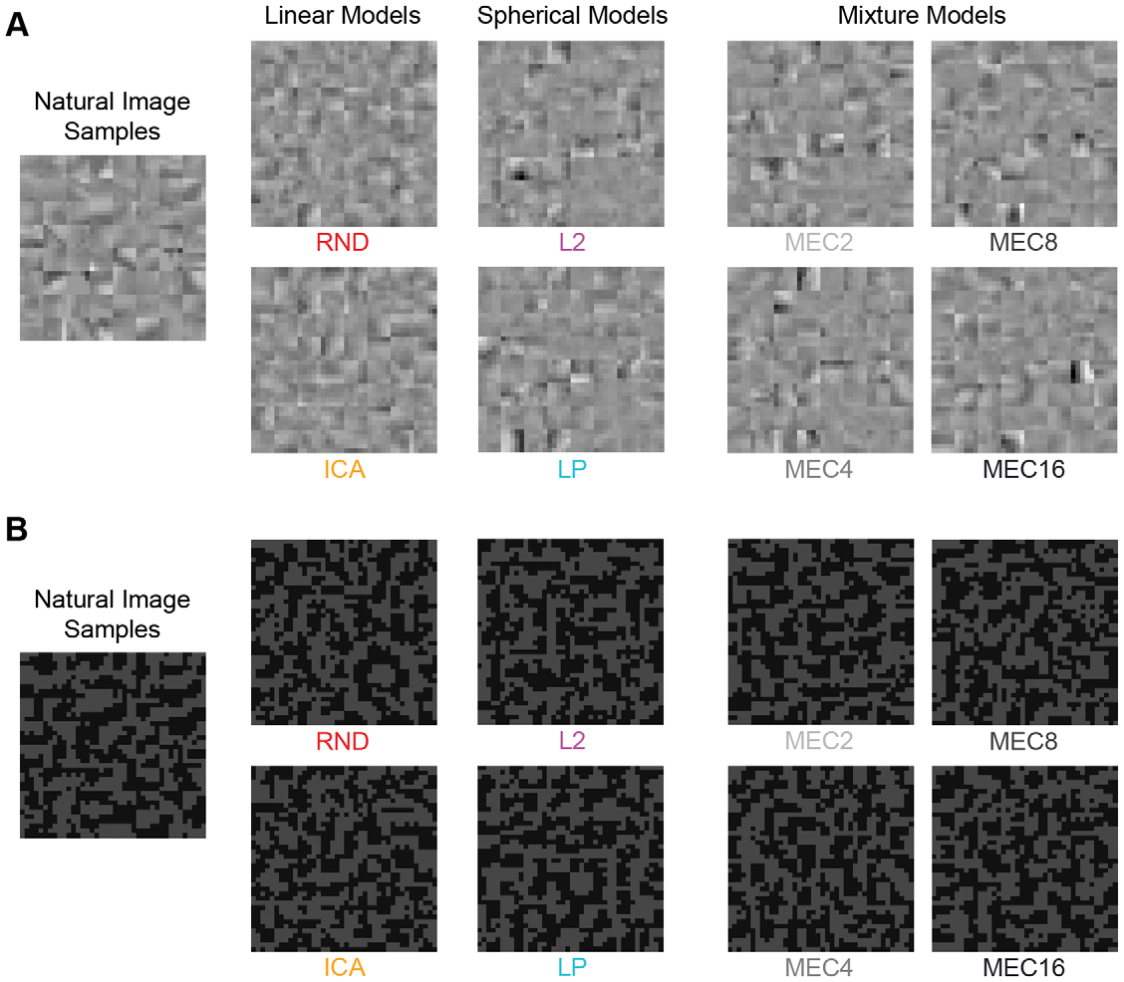
Experiment 4 high contrast stimuli. To focus on regions of natural images containing shape information, we automatically selected high contrast natural image patches for use as stimuli. **A.** Grayscale stimuli for the 8 models we tested: RND, ICA, L2, LP, MEC2, MEC4, MEC8, MEC16. **B.** The binary version of **A** where the number of on and off pixels are held equal. On any only trial, the observer viewed only one set of natural image samples and one set of samples from a single model.

To avoid homogeneous patches lacking shape content, we limited our natural samples to high contrast image patches. In a pilot binary experiment where we considered all possible natural image patches, we found that the number of homogeneous patches is a highly prominent cue, so we wanted to remove it from this experiment and focus instead on shape information located in the heterogenous regions of natural images. However, it turned out that using only high contrast patches increases the difficulty of the task greatly, so we again used unlimited presentation times as in Experiment 3. To account for the increased difficulty of the high contrast stimulus set, we measured performance for grayscale (unperturbed) stimuli in addition to the binary version. A set of high contrast grayscale stimuli is shown in Figure 11A with the binary version in Figure 11B.

All experimental parameters and models were the same as in Experiment 3, except that we tested all four MEC models with 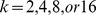 mixtures. MEC models were blocked into two sessions, one for the grayscale high contrast patches, the other for the binary version. The other models were analogously blocked. Seven subjects participated. Each completed 36 test trials per model per patch size per session for a total of 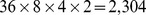 trials.

**Figure 12:**
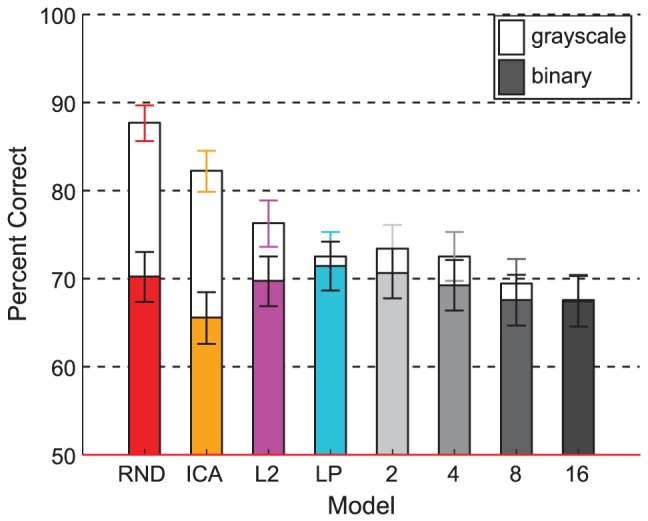
Experiment 4 results. Discriminability estimates with 95% binomial confidence intervals are shown by model in order of increasing likelihood where data are pooled over subjects and patch sizes (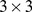, 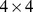, 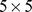, and 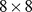). Seven subjects participated, and each performed 36 test trials per model per patch size per condition, so each data point is based on 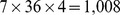 trials. Unfilled bars are for the grayscale high contrast stimuli, and filled bars the binary version. Within the range of the error bars, the estimates for the grayscale stimuli followed the same ordering as in Experiment 1, yet the data for the binary stimuli show no ordering.

Results are shown in Figure 12, where we plot discriminability estimates with 95% binomial confidence intervals for each model in order of increasing likelihood, with trials pooled over patch sizes. Unfilled bars show estimates for grayscale stimuli and filled bars for binary stimuli. Grayscale stimuli led to the same discriminability ranking of the models as in Experiment 1. In the binary condition, on the other hand, the model ordering disappeared. The above chance performance indicates that all models failed to capture the binary shape cues perfectly for the observers.

Again, using the learning test reported in Experiment 1, we analyzed the data for each model separately for the two conditions with data pooled over subjects and patch sizes. There were no significant effects of learning.

## Discussion

Several psychophysical studies have measured texture discrimination in terms of statistical constraints used to generate artificial stimuli [36]–[40], [46]–[49] and have established an extensive description of local image statistics to which the human visual system is sensitive. One study [50] has linked sensitivity to synthetic textures with the informativeness of natural image regularities as well. In the current study we use probability density function models of natural images to directly measure sensitivity to statistical regularities present in natural images. Previous work with such models largely focuses on the link between natural image statistics and neurophysiology [3]–[11], [31]–[33] although one study evaluates the perceptual redundancy of the independent components analysis model [51]. In this work we evaluated the link between perceptual sensitivity and a variety of probability density function models of natural images.

After testing a series of natural image models from one capturing only second-order correlations (RND) to one among the current state-of-the-art in capturing higher-order correlations (MEC, [33]), we found that human observers achieved above chance performance in most cases (Figure 5) and that discriminability was worse for models with higher likelihood, i.e., models that captured more natural image regularities. However, even for the model with the highest likelihood, observers were well above chance to discriminate natural image regularities from model regularities for patches 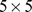 pixels (approximately 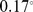 during natural viewing) in size or larger, which suggests that the human visual system possesses a surprisingly detailed knowledge of the natural image distribution, at least in comparison to the models currently studied in the machine learning community.

There were a number of reasons we might not have found such high levels of performance. The stimulus patches corresponded to very local image regions, and the pixel quantization was clearly visible, which could have masked some of the low spatial frequency content of the stimuli. Furthermore, the natural image dataset [41] is likely to include some images with significant blur whenever there was a limitation in the depth of field. To the extent that these kinds of issues affected our data, they could only underestimate human potential on the task, so the impressive levels of above chance performance we report are only lower bounds.

The second important conclusion relates to the model ordering in terms of discriminability. To explain the significance of this result in terms of understanding the human visual system, we need to return to the idea of the natural image distribution, which we alluded to in the Introduction with Figure 1. The distribution of all possible natural images has a particular density that differs greatly from uniform because natural images have a high degree of correlations. Model likelihood describes how well a model captures the true density. A separate question is how sensitive human observers are to the natural image distribution. The human visual system need not be sensitive to all information in images and thus may be optimized only for a subset of regularities that are perceptually relevant. In this case, if higher likelihood merely indicates that a model captures more regularities regardless of perceptual relevance, models with high likelihood need not lead to more natural looking samples than low likelihood models. However, for all models tested here, human performance was worse with increased model likelihood. We found this ordering relationship in all experiments where luminance values were unperturbed (Figures 6A, 6B, 8B, 10C, and 12 unfilled bars). We regard this ordering as evidence that the visual system is biased for processing natural images.

Our discrimination task constitutes a high dimensional classification problem. Each experiment was run in a single session less than 90 minutes in duration, and in all cases we found very little or no evidence of learning during the test trials. The fact that observers could learn this task so quickly during the few training trials indicates a bias of the system for processing natural images. In machine learning, the “no free lunch” theorem [35] states that all classification algorithms perform the same on average over all tasks. Put another way, the performance of a classification algorithm–-including those of human observers–-on our task reflects how biased it is for this particular task. While the pattern of performance provides evidence for the existence of a bias, it does not provide specific feedback about how the bias is implemented by the visual system. In the three cue identification experiments, we examined which natural image features were prominent cues to the discrimination task.

We can draw two clear conclusions about the perceptual importance of some of the model features.

The first conclusion relates to the importance of filter shapes. We tested two pairs of models that differed only in filter shape: a random second-order model and the independent components analysis model (RND vs. ICA) and the 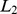-spherically symmetric model using random filter shapes and the 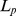-spherical model using the ICA filters (L2 vs. LP). Even though a general proportionality between discriminability and model likelihood does not exist (only an ordering relationship), the previously reported small effect of filter shape on likelihood [8], [43] was mirrored by very small differences in discriminability here. As shown in Figures 6A and 6B, the differences from RND to ICA and from L2 to LP are very small, indicating that using a linear transformation with oriented filters translates into a very small perceptual benefit. This result implies that the oriented filters of the independent components analysis model make only a small improvement over a pink-noise like representation in capturing perceptually prominent natural image features.

The second conclusion is that spherical models do not fully reproduce local luminance histogram variations sufficiently (Figure 8A long dashed lines) even though they are meant to capture contrast fluctuations [7]–[10]. Overall, the most difficult model to discriminate from natural images was the mixture of elliptically contoured distributions model, which apparently reproduced the luminance histograms sufficiently but failed to capture the structural patterns in natural images sufficiently at the largest sizes we tested.

### Local structure information

Our results indicated that luminance histogram features are highly informative about model versus natural image identity for most of the models we tested. In Experiments 3 and 4, we aimed to “partial out” these cues and evaluate model efficacy with respect to structural information instead. Previous studies of human sensitivity to local shape structure show that particular fourth-order correlations in binary images are perceptually salient [39], [40], [49] and correspond to informative features of natural images [50]. We wanted to examine the extent to which the models we tested capture any kind of perceptually prominent structural information at the patch sizes presented.

In Experiment 3, where we removed the contrast fluctuation cue, the ordering of the models in terms of discriminability dramatically changed. The originally large difference from our second-order model (RND) to the spherical models capturing higher-order correlations (L2, LP) disappeared, and all three models were the same in terms of difficulty (Figures 10B). This suggests that the main advantage of the spherical models has little to do with capturing the structural or shape content of natural images. Rather, when this result is taken together with the results of Experiment 2, where L2 and LP were more difficult than RND with luminance histograms as the only cue (Figure 8), it is clear that the main advantage of spherical models is due to better preservation of contrast fluctuations. What was more surprising was that the ICA model became the most difficult model to discriminate from natural images (Figures 10B), suggesting that the shape of the ICA filters offers some advantage over random filters although they are not perfect, as indicated by above chance performance.

In Experiment 4, we removed all luminance histogram cues by using binary image patches as stimuli (where patches were automatically selected to contain spatial variations). Thresholding the luminance values preserves the shape of the luminance contours. When this kind of shape information is the only cue, all models were equally difficult although observers were still above chance (Figure 12 filled bars), meaning that they can make use of such shape cues. Even though the percent correct for ICA was again lower than the other models, this difference is not significant here. Discriminability was not affected by binarization for LP and all higher likelihood models (overlap of filled and unfilled bars in Figure 12), which suggests that the luminance contour shapes are likely to be one of the main cues used to discriminate these models from natural images. We take these results as an indication that the shapes of the luminance contours preserved after binarization are an important perceptual feature of natural images. Elder has demonstrated their perceptual informativeness using a different technique in which he reproduced the appearance of photographic images very well from only such contours and local contrast values [52]. Our results suggest that none of the common grayscale natural image models captures these contour statistics sufficiently and that higher likelihood models are no better with it than a random second-order model.

### Studying visual sensitivity to natural image regularities

Studying sensitivity to natural image regularities is a challenging pursuit for several reasons, not least of which is their high-dimensional complexity. One approach is to focus on a particular aspect of natural images, measure its distribution, and examine whether the visual system is biased for the empirical distribution. Girschick and colleagues [53] have taken such an approach to study the visual system's knowledge of local orientation statistics in natural images. Other approaches rely on generating stimuli with controlled natural image features. The more classical technique has been to use the Fourier transform to examine sensitivity to higher-order natural image correlations via phase quantization or scrambling in large images, e.g. [19]–[25], and a more recent technique is to use a texture synthesis model, such as the Portilla-Simoncelli model [29], which can represent a wide range of natural textures very convincingly and whose parameters can be interpreted in terms of neural responses. Psychophysical studies using the Portilla-Simoncelli model have advanced our understanding of peripheral visual processing [54],[55], and have uncovered physiological properties of early visual cortex [56].

We used a new technique to generate stimulus images. Our approach is to selectively randomize the content of true natural images within the assumptions of a probabilistic natural image model. The primary difference between our approach and previous ones is that our stimuli are explicitly constructed to be equally probable for a given probabilistic image model, so it allows us to test the model assumptions. Furthermore, by using models whose likelihoods have been computed, we can directly relate performance to the degree of regularities captured by the model.

### Natural image model evaluation

It is an open question how best to evaluate probabilistic models of natural images, and a variety of quantitative analyses have been used previously, including reconstruction error, multi-information and likelihood evaluation (e.g. in [8]). Likelihood is proportional to the amount of regularities a model captures, yet the total amount of regularities present in the natural image distribution is not known, nor was it known whether likelihood relates to perceptual measures of model efficacy. The results of our experiments show, however, that likelihood seems to have good predictive power about perceptual relevance.

While many machine learning studies have based their model comparisons on *ad hoc* judgments about the perceptual resemblance to natural images, our paradigm provides a rigorous tool for model evaluation and comparison: psychophysical discriminability measures, which vary from chance (perfect model) to ceiling (significant model failure). Furthermore, the paradigm can be used to measure model performance at capturing particular natural image features (e.g. our cue identification experiments).

## Methods

### Ethic statement

The experiments were approved by the Ethics Commission of the Medical Faculties of the Eberhard Karls University and the University Clinics of Tübingen. All subjects gave informed consent prior to the experiment.

### Subjects

Subjects were adults with normal or corrected-to-normal vision. All subjects were naive except author HEG who participated in Experiment 1 session 1, and Experiments 2–4.

### Apparatus

Stimuli were displayed on a linearized Siemens SMM 21106 LS 21-inch CRT monochrome display, which had a maximum luminance of 423 cd/m^2^, in a dim room. A forehead bar and chinrest were used to fix the viewing distance at 90 cm. Experiments 1 and 2 used a Cambridge Research Systems Visage graphics controller with a 14-bit grayscale resolution, Cedrus RB-530 response box, and were programmed using the Cambridge Research Systems VSG toolbox for MATLAB. Experiments 3 and 4 used a custom DATAPixx controller with 16-bit grayscale resolution, the 5 button RESPONSEPixx response box, and were programmed using the Psychophysics Toolbox for MATLAB [57], [58].

### Natural image samples

All natural image sampling and modeling was performed using the Natural Image Statistics Density Estimation Toolbox (nisdet) [59]. For each patch size (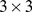, 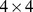, 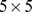, and 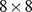 pixels), a set of 64,000 natural image patches, 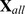, were sampled uniformly both across and within the images of the van Hateren natural image database [41]. 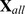 is an 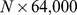 matrix where 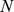 is the number of pixels in the patch. We stored the natural logarithm of the individual pixel values in 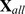. We centered 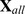 by removing the row mean from each entry in each row and the column mean from each entry in each column and then scaled the result such that the 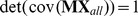, where 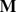 is a matrix that projects out the DC component using a QR decomposition, which makes whitening a volume conserving transform [8].

We preprocessed the data with filter matrices 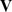 of the form 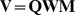, where 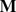 is the aforementioned matrix that projects out the DC component, 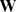 is a whitening matrix, and 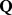 is an orthogonal matrix (i.e. 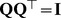). While we kept 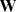 and 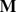 fixed, we varied 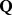 to determine the actual filter shapes. Note that 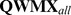 is white for any orthogonal matrix 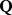. Each filter matrix can be inverted to define a complimentary synthesis matrix 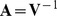 such that 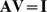.

We used two types of orthogonal matrices: 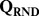, a random orthogonal matrix, and 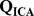, the independent components analysis model basis. 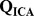 was learned using the fastICA [60] algorithm to initialize the filter shapes. We then optimized them via a gradient ascent on the log-likelihood of a factorial model with exponential power distributed marginals [8]. Below, the subindex of 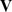 denotes which 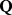 was used.

For each mixture of elliptically contoured distributions model, we first clustered the natural image data into 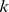 clusters using k-means. Then we calculated the inverse square root covariance matrix of each cluster, 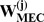, where 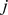 indexes the cluster number from 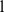 to 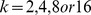 depending on the number of mixtures in the model.

### Model samples

To generate model samples, 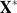, we start with 64 image patches, 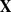, randomly sampled from 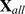. (In a single experimental session, different 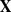 are sampled on every trial for each model patch size combination, but the same superset of all 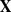 used to test one model patch size combination are used to test all other models at that patch size.) We use the following general formula: 1) transform 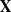 to the coordinate system of the model using the appropriate filter matrix 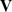: 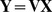, 2) apply the model assumptions to 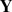 to obtain a new 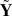, 3) transform back to pixel space using the appropriate synthesis matrix 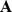: 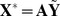.

The kind of shuffling applied in step 2 is determined by the model assumptions. We use two types of such shuffling. The first type applies an independence assumption to the data and shuffles the non-DC coefficients within each coordinate separately across all samples in 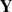. The second type applies a symmetry assumption to the data and permutes the non-DC coefficients separately within each patch in 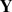. Because the norm of a patch is permutation invariant, this permutation preserves the norms of the patches in the whitened space.

To create RND samples and ICA samples, we apply the independence assumption shuffling procedure, using 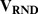 and 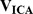 respectively.

To create L2, LP, and MEC samples, we apply the symmetry assumption shuffling procedure. For L2 samples, we use 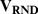, the second order basis. To create LP samples, we use 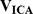 since ICA is the optimal basis for LP [10]. For MEC samples under an MEC model with 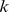 mixtures, we first assign each patch in 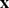 to one of the 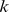 clusters of the model by evaluating the patch's maximum likelihood cluster membership. Then for all patches in the 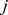-th cluster we use the corresponding 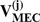 analysis matrix of the maximum likelihood cluster.

The Fourier phase scrambled samples were created using a different approach. IPS samples were created by storing the amplitude spectra of the patches in 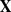 and combining them with random phases before inverse Fourier transforming back to image pixel space. GPS samples were created by storing both the amplitude and phase spectra of the patches in 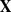 and then reassigning the individual phase spectra randomly to different patches in 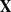 before inverse Fourier transforming back to image pixel space.

The resulting matched samples, 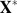, where then tiled tightly into a square texture as were the samples of 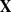. For grayscale conditions, the gray values of the two textures taken together were normalized from the range 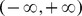 to 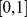 to utilize the full gamut of the CRT monitor. For the binary textures of Experiment 4, the gray values of each patch in 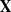 and 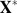 were thresholded such that the resulting binary patch had equal numbers of white and black pixels (4 white pixels for 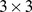 and 12 white pixels for 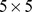 pixel patches). Binary textures did not utilize the full gamut of the monitor as this level of contrast was uncomfortable to view for extended periods. Instead, luminance was lowered such that white was approximately 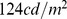.

### Experiment 1

In Experiment 1 we used a two-alternative forced choice task to measure the discriminability of textures of natural image samples from textures of model samples. In the first session with 16 subjects, RND, ICA, L2, LP, IPS, and GPS were used to generate stimuli at patch sizes 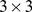, 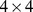, and 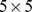 pixels. In session two with 12 subjects, MEC 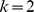, MEC 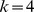, MEC 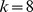, and MEC 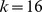 were used to generate stimuli at patch sizes 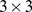, 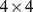, 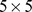, and 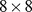 pixels. In both experiments, observers first completed 20 training trials with 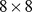 pixel patches for each model before starting the experimental session with 30 test trials per model per patch size. Trials were grouped in small runs by model in order of decreasing image patch size. The ordering of the models across runs was randomized.

Texture sizes were 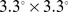 for 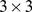 pixel patches, 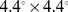 for 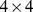 pixel patches, 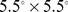 for 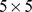 pixel patches, and 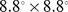 for 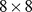 pixel patches. The textures were presented side by side for 3000 msec with additional 200 msec sinusoidal ramps on and off. There was a 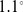 blank space between the two textures. The true natural samples appeared on the right and left sides with equal probability. After stimulus extinction, the subject reported which side contained the true natural image samples and was provided immediate feedback by an auditory tone. If the incorrect texture was chosen, the stimulus was shown again for 3900 msec with the correct texture highlighted by a white frame.

One subject completed 4,032 trials of a four-alternative forced choice version of the experiment, where each stimulus included four textures: one contained the true natural image samples, 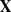, and the other three contained model generated samples, 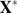, each matched statistically to 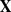 but different in exact appearance. The four textures were arranged in an invisible 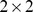 grid on the screen with 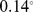 blank space separating them. As in the main experiment, the task was to select the one texture made of natural samples, which appeared at each location in the grid with equal probability. We used this design as it is the preferred method for naive observers [61]. The subject completed four sessions with RND, ICA, L2, LP, IPS, and GPS, and one session with the MEC models. Each session contained 36 test trials per model per patch size tested, which were 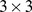, 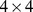, 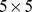, and 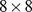. Because this version of the experiment contains much more visual information to inspect on each trial, we allowed the subject to view the stimuli for as long as needed but instructed that the response should be made as quickly as possible without sacrificing accuracy. Feedback screens were shown for 5400 msec. We adjusted the texture sizes so that four could be presented simultaneously and so that the texture sizes would be approximately the same to facilitate faster responses. The texture sizes were 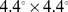 for 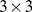, 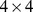, and 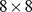 pixel patches and 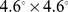 for 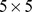 pixel patches.

### Experiment 2

Experiment 2 measured sensitivity to luminance histogram cues in natural image samples. It was identical in design to the two alternative forced choice version of Experiment 1 except that we scrambled the pixels of the textures as a final post-processing step before painting them to the screen. We excluded MEC 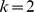, MEC 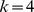, and MEC 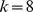, and the experiment was run in two one-hour sessions separated by condition. In the first condition we permuted the pixels globally within each texture to produce “global scrambles.” In the second condition, we permuted pixels within each sample separately to produce “sample scrambles.” Three subjects participated.

### Experiment 3

In Experiment 3, we used a two alternative forced choice task to measure sensitivity to the grayscale shape information in natural image samples separately from the contrast fluctuation cue. Because MEC, IPS, and GPS perfectly captured the contrast fluctuation cue in Experiment 2, we excluded them and measured discriminability only for RND, ICA, L2, and LP at patch sizes 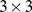, 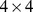, 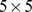, and 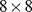 pixels. Nine observers participated, each contributing 36 test trials per model per patch size.

We matched the distribution of gray value norms in the model samples, 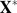, to the distribution of gray value norms in the natural image samples, 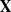, on a trial-by-trial basis, where the gray value norm of a patch is the Euclidean length of the vector of pixel values. Because the norms are measured on patches with zero mean, they are related to r.m.s. contrast. Patches whose pixel values vary greatly across the patch (high contrast) have large norms, and homogeneous patches have much lower norms independent of the mean gray value. Our procedure was the following: 1) compute the gray value norms of all patches in 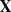 and in 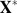 2) sort the norms of 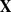 in increasing order, 3) sort the patches of 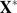 in increasing order of their norms, 4) scale the 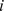-th patch in 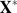 to have the value of the 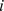-th entry in the sorted norms of 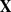, 3) shuffle the patch positions within 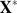. For RND and ICA samples, we scaled each sample in 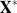 by a gamma random variable prior to step 1. Gamma distribution parameters had been optimized beforehand via simulations to minimize perturbations in pixel covariances.

Because the task is more difficult when the contrast fluctuation cue is removed, we allowed subjects to view the stimuli as long as necessary. However, we encouraged them to respond as quickly as possible without sacrificing accuracy and also used different texture sizes than in Experiments 1 and 2, so that the stimuli would be roughly the same size on every trial to facilitate faster visual processing. The texture sizes were 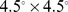 for 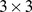 and 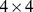 pixel patches and 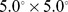 for 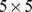 and 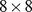 pixel patches. We measured discriminability for the unperturbed stimuli as well under the same timing and size parameters. The experiment therefore had two randomly interleaved conditions, one for the unperturbed stimuli, the other for the contrast distribution matched stimuli. All other aspects of the experimental design were identical to the two alternative forced choice version of Experiment 1.

### Experiment 4

In Experiment 4, we used a two alternative forced choice task to measure sensitivity to the cues present in binary images. Only natural image patches above the median patch contrast value were used as stimuli. Natural patches, 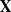, of patch size 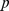 were therefore selected only from the upper half (in terms of contrast) of the corresponding dataset 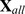. The discrimination task was more difficult with these high contrast stimuli than with the stimuli of Experiment 1, so we therefore used the timing and textures sizes of Experiment 3. We measured discriminability for all models with grayscale unperturbed stimuli in addition to the binary version. The experiment was two one-hour sessions: session 1 for RND, ICA, L2, and LP, and session 2 for the four MEC models. Each one-hour session consisted of two shorter sessions, the first was the grayscale version, and the second was the binary version. Seven subjects participated, each contributing 36 test trials per model per patch size (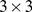, 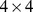, 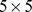, and 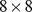 pixels). All other design details were identical to the two alternative forced choice version of Experiment 1.
